# Genetic polymorphisms of rs9313422 G>C and rs41297579 G>A at the promoter of *TIM‐1* gene contribute to the risk of community‐acquired pneumonia in children

**DOI:** 10.1002/jcla.23095

**Published:** 2019-12-04

**Authors:** Yang Liu, Hong‐Bo Xu

**Affiliations:** ^1^ Department of Pediatrics First Affiliated Hospital of Yangtze University Jingzhou China; ^2^ Neonatal Department Maternal and Child Health Hospital of Jingzhou City Jingzhou China

**Keywords:** children, community‐acquired pneumonia, polymorphism, TIM‐1

## Abstract

**Objective:**

To investigate the association of genetic polymorphisms of rs9313422 G>C and rs41297579 G>A at the promoter of *TIM‐1* gene with the risk of community‐acquired pneumonia (CAP) in children.

**Methods:**

A total of 112 children with CAP were included as the case group. Another 120 healthy children were enrolled as the control group. Polymerase chain reaction‐restriction fragment length polymorphism (PCR‐RFLP) was applied for the genotyping of rs9313422 G>C and rs41297579 G>A in the promoter region of *TIM‐1*.

**Results:**

rs9313422 G>C was related to the risk of CAP in children under codominant model, dominant model, recessive model, and allele model. Besides, the A allele of rs41297579 G>A could increase the risk of CAP in children. Besides, the haplotype GA (rs9313422‐rs41297579) and GG reduced the risk of children CAP, while haplotype CA had an elevated risk. rs9313422 G>C and rs41297579 G>A polymorphisms were both associated with the severity of CAP in children, and the rs9313422 G>C was also related to the ICU admission rate. In addition, patients carried with the mutant homozygotes of rs9313422 G>C and rs41297579 G>A showed higher levels of white blood cell (WBC), procalcitonin (PCT), and C‐reactive protein (CRP) than the wild type and heterozygous genotypes carriers.

**Conclusion:**

rs9313422 G>C and rs41297579 G>A polymorphisms in the promoter region of *TIM‐1* could increase the risk of CAP in children and showed a relation with inflammatory responses and severity.

## 1 INTRODUCTION

In general, pneumonia is an inflammatory condition of the lung parenchyma, affecting the alveoli with the infection of pathogenic microorganisms, immune injury, allergies, drugs or other physical, and chemical factors,^1^ which was also one of the leading causes of death among children under 5 years of age.^2^ While, the acute infection of the pulmonary parenchyma, developing outside the hospital or diagnosing within 48 hours of admission to the hospital, was credited as community‐acquired pneumonia (CAP), which was common in children.^3^, ^4^ According to the epidemiological investigation, CAP was believed as one of the most severe acute respiratory infections (ARI), approximately accounting for 80% of all deaths suffering from this disease,^5^ which was more frequent in developing countries.^6^ Moreover, the incidence rate of CAP in children was nearly 22%, and there were about 55,000 children deaths every year caused by CAP in China.^7^ Although the pathological mechanism of CAP has not been clearly elucidated, many researchers have focused their attentions on genetic factors in CAP.^8^ For instance, the GG genotype and G allele of −174 G/C in *IL‐6* (an anti‐inflammatory cytokine) had a close relation to the susceptibility of CAP in Egyptian children, as suggested by Zidan et al^9^ The human T‐cell immunoglobulin mucin (*TIM*) gene family, located on the human chromosome 5q23‐35, contains three members, including *TIM‐1*, *TIM‐3,* and *TIM‐4*,^10^ which was shown to be relevant to the immune dysfunction, consequently inducing various diseases, such as autoimmune diseases, allergic diseases, chronic viral infections (CVI), immune rejections, and even tumors.^11^ As the first identified gene, *TIM‐1*, also known as *HAVCR1*, was responsible for regulating immune responses and maintaining immune homeostasis, which had relations to several human inflammatory diseases,^12^ and took part in the occurrence and progression of pneumonia. In the study by Fan et al, patients with mycoplasma pneumoniae pneumonia (MPP) were statistically different from healthy controls in the tilters of TIM1, and blocking TIM1 expression may reduce the risk of MPP‐induced damages in heart and liver.^13^ In recent years, many scholars have focused on the effects of *TIM‐1* gene polymorphisms and diseases. For instance, the −1637A > G and −232A > G single nucleotide polymorphisms (SNPs) in the *TIM‐1* gene were demonstrated to be linked to the susceptibility to rheumatoid arthritis (RA) by Xu et al^14^ Meanwhile, Kim et al showed that the insertion of a 6‐amino‐acid in *TIM‐1* (157insMTTTVP) had some impact on a Hepatitis A virus (HAV)‐induced severe liver disease.^15^ More importantly, the two common SNPs (−1454 G>A (rs41297579) and −416 G>C (rs9313422) within *TIM‐1* were associated with respiratory diseases, like asthma,^16^, ^17^ but it has not been explored whether they have something to do with the susceptibility to CAP. Given the above, 112 children with CAP and 120 healthy controls were recruited as the subjects of study, and polymerase chain reaction‐restriction fragment length polymorphism (PCR‐RFLP) was used for the genotyping, to investigate the relationship between SNPs including rs9313422 G>C and rs41297579 G>A in the promoter region of *TIM‐1* and the risk of in children with CAP.

## 2 MATERIALS AND METHODS

### 2.1 Ethics statement

This study got the approval of the Ethics Committee in Maternal and Child Health Hospital of Jingzhou City and strictly followed the Helsinki declaration.^18^ The guardians of all patients signed an informed consent prior to study and agreed to the information collection and blood collection in clinical trials.

### 2.2 Subjects of study

From May 2015 to May 2017, a total of 112 children with CAP in Maternal and Child Health Hospital of Jingzhou City were included as case group. All CAP patients met the diagnostic criteria of CAP created by the pediatric infectious diseases society and the infectious diseases society of America 19 as follows: (1) newly acquired cough and sputum or pre‐existed respiratory tract symptoms aggravating due to purulent sputum, with or without chest pain; (2) fever; (3) signs of pulmonary consolidation and/or moist rale; (4) white blood cell count > 10 × 10^9^/L or < 4 × 10^9^/L, with or without nucleus shift to left; and (5) invasive shadow in patches detected by chest X‐ray, with or without pleural effusion. Clinical diagnosis can be identified when patients met one criteria of (1)‐(4) and (5). And the pneumonia of patients can be identified as community‐acquired if they had no history of hospitalization during the 2 weeks prior to admission. In addition, the control group consisted of another 120 healthy children who underwent physical examination during the same period in our hospital.

### 2.3 Inclusion and exclusion criteria

All subjects were unrelated Chinese (Han nationality), who were newly diagnosed and treated. The exclusion criteria were as follows: patients who combined with respiratory diseases, such as tuberculosis, non‐infectious interstitial lung diseases, pulmonary edema, pulmonary atelectasis, pulmonary embolism, pulmonary eosinophilia, and pulmonary vasculitis, and particularly asthma; patients who were with a history of surgery or operations, serious dysfunction of heart, liver, kidney, lung or other organs, immunodeficiency, tumors or a history of tumor, hematologic disorders, severe damage in central nervous system, diabetes mellitus, obesity, or other infectious diseases; patients who exhibited poor compliance in the blood collection process; and patients who were unsuitable for intravenous blood sampling.

### 2.4 Isolation of genomic DNA

Fasting peripheral sample (3 mL) was extracted from peripheral venous blood of all subjects, which were anti‐coagulated with the addition of EDTA and centrifuged for 10 minutes at the rate of 1500 *g*. Next, DNA extraction kit (Takara Company) was used to extract the whole blood DNA followed by the determination for the purity by a DNA concentration detector (NANODROP2000, Thermo Company), with the A260/A280 ratio between 1.6 and 1.8. The DNA extracted was preserved at −20°C in a refrigerator.

### 2.5 Genotyping

Polymerase chain reaction‐restriction fragment length polymorphism (PCR‐RFLP) was applied for the genotyping of − 1454 G>A (rs41297579) and −416 G>C (rs9313422).^11^ The primer sequences were listed as follows: rs9313422 G>C: Forward: 5’‐GCATGTTGTACAGGAGCATGA‐3’, Reverse: 5’‐GCAGACAGGCTGGTTGGTACC‐3’ and rs41297579 G>A: Forward: 5’‐ CAGGTTGGTCTCAAACTCCTT‐3’, Reverse 5’‐TTCCAAGGAGGCAGTGGTGG‐3’. The PCR reaction was performed in a 25 µL total volume (2.5 L 10 × PCR buffer, 1.5 mmol/L MgCl_2_, 0.15 mmol/L dNTPs, 0.5 μmol/L each primer, 100 ng genomic DNA, and 2U of Tag DNA polymerase) under the conditions as follows: 94°C for 4 minutes, 35 cycles of 94°C for 30 seconds, 58‐65°C for 45 seconds, 72°C for 45 seconds, and finally extending at 72°C for 5 minutes using the BioRad MyCycler Thermal Cycler (BioRad Laboratories). Besides, the products of PCR (3.5 μL) were purified with a PCR purification kit (Takara Biologicals), followed by digestion with corresponding locus specific restriction enzymes (Takara Biologicals), including *Taq*I (rs9313422 G>C) or *Msp*I (rs41297579 G>A) in 10 μL solution for 2‐3 hours. The digested PCR products were added into 2% agarose gel (containing ethidium bromide) for electrophoresis. About 10% of the samples were randomly sent for DNA sequencing.

### 2.6 Statistical method

Data processing was performed with the statistical software SPSS 21.0 (SPSS Inc). Hardy‐Weinberg equilibrium was applied to evaluate the representativeness of rs9313422 G>C and rs41297579 G>A in the Case and control groups. Logistic regression analysis was used to calculate the odd ratio (OR) and 95% confidence intervals (CI), so as to estimate the strength of association between the mutation of polymorphic loci and CAP in children. Enumeration data were presented by rate or ratio, which was compared by using Chi‐Square (*χ* ^2^) test. Measurement data were presented by mean ± standard deviation ($\bar{x}$±s). Comparison between two groups was analyzed by Student's t‐test. Comparison among multiple groups was conducted by using one‐way ANOVA followed by using Tukey's post hoc test. A two‐tailed *P* value < .05 indicated that the difference was of statistical significance.

## 3 RESULTS

### 3.1 Baseline characteristics of subjects

The baseline characteristics of 112 CAP patients in the case group were shown in Table 1. There are 64 males and 48 females with the average age of 57.55 ± 37.17 months (3‐118 months). In addition, healthy subjects in the control group were consisted of 62 males and 58 females with the average age of 57.00 ± 31.63 months (4‐110 months). Obviously, no statistical difference was found between the case group and the control group in age and gender (both *P* > .05).

**Table 1:** Baseline characteristics of 112 CAP children

Characteristics	n = 112
Age (mo)	57.55 ± 37.17 (3‐118)
Gender	
Male	64
Female	48
Identified pathogens	
Bacterial	11
Viral	9
Bacterial and viral	3
Yeast	3
Unknown	86
Pneumonia severity	
Mild	37
Moderate	41
Severe	34
WBC (×10^9^/L)	
>10	100
≤10	12
PTC (ng/mL)	
>2	52
≤2	60
CRP (mg/L)	
>25	73
≤25	39
ICU admission	
No	29
Yes	83
Mortality	
No	106
Yes	6

### 3.2 Genotyping and sequencing of rs9313422 G>C and rs41297579 G>A in the promoter region of *TIM‐1*

**Figure 1:**
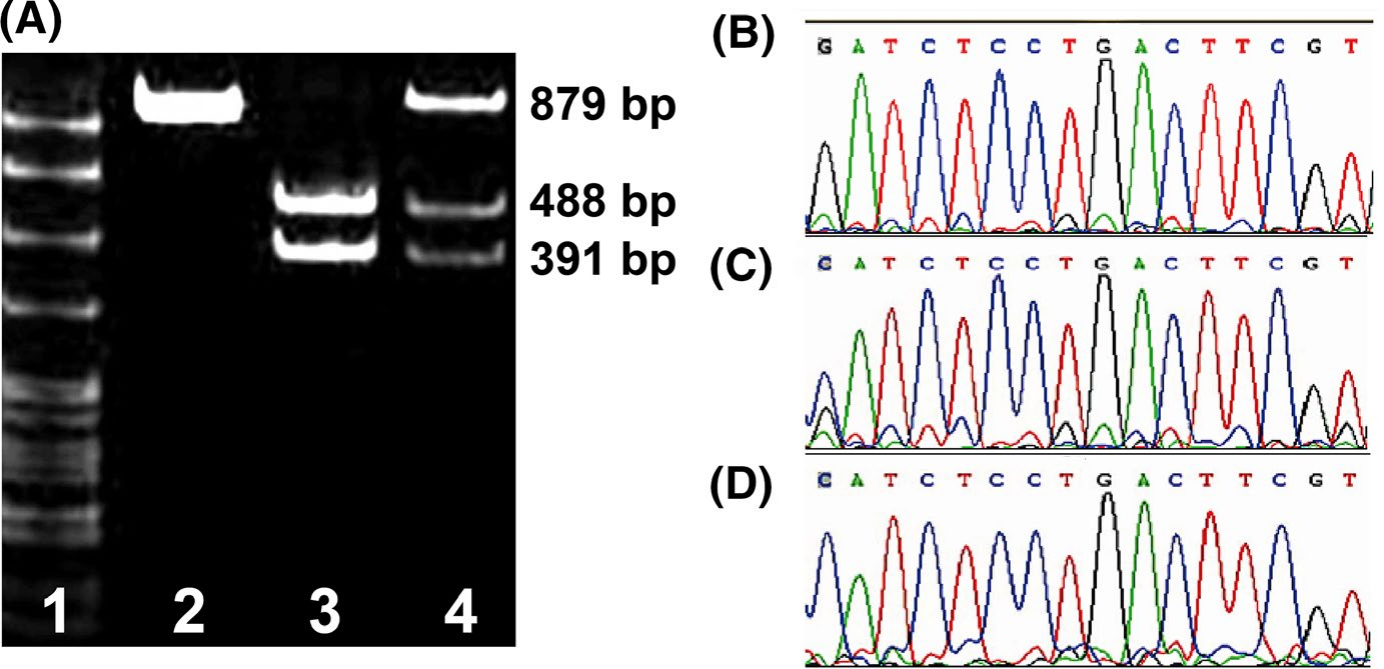
Genotyping and sequencing of the rs9313422 G>C polymorphism of the *TIM‐1* gene. Note: A, Genotyping of *TIM‐1* rs9313422 G>C polymorphism by PCR‐RFLP; lane 1, 50‐1000bp DNA marker; lane 2, CC genotype (a 879bp fragment); lane 3, GG genotype (two fragments: 391bp and 488bp); lane 4, GC genotype (three fragments: 391bp, 488bp, and 879bp); B‐D, DNA sequencing of *TIM‐1* rs9313422 G>C polymorphism, with GG genotype having only one G peak (B), GC genotype having two peaks (G peak and C peak) (C), and CC genotype having only one C peak (D)

**Figure 2:**
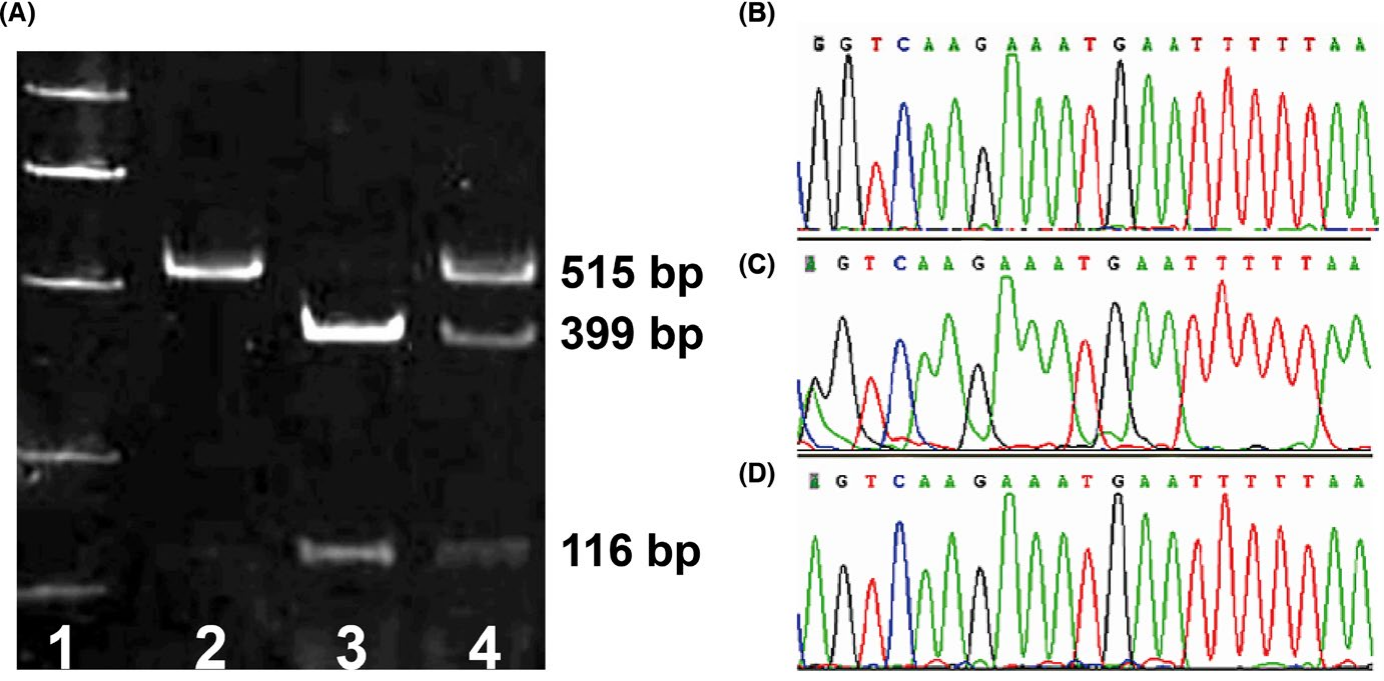
Genotyping and sequencing of the rs41297579 G>A polymorphism of the *TIM‐1* gene. Note: A, Genotyping of TIM‐1 rs41297579 polymorphism by PCR‐RFLP; lane 1, 50‐1000bp DNA marker; lane 2, AA genotype (a 515bp fragment); lane 3, GG genotype (two fragments: 116bp and 399bp); 4, GA genotype (three fragments: 116bp, 399bp, and 515bp); B‐D, DNA sequencing of TIM‐1 rs9313422 G>C polymorphism, with GG genotype having only one G peak (B), GA genotype having two peaks (G peak and A peak) (C), and AA genotype having only one A peak (D)

The PCR amplification product of rs9313422 G>C was 879bp in length. The digestion products showed three genotypes, including CC (a single uncuttable fragment of 879bp), GG (two fragments of 488bp and 391bp), and GC (three fragments of 879bp, 488bp, and 391bp), as shown in Figure 1A. After DNA sequencing, it was confirmed that GG genotype had only one G peak, CC genotype had only one C peak, while GC genotype had both a G peak and a C peak (Figure 1B‐C). In addition, the PCR amplification product of rs41297579 G>A was 515bp, and *Msp*I cut the 515bp PCR product into two fragments of 399bp and 116bp length. A single 515bp band exhibited the presence of AA genotype; fragments 399bp and 116bp indicated the presence of the GG genotype and three fragments of 515bp, 399bp, and 116bp displayed the presence the GA genotype (Figure 2A). Meanwhile, DNA sequencing showed that GG genotype had only one G peak, AA genotype had only one A peak, while GA genotype had both a G peak and an A peak (Figure 2B‐C).

### 3.3 Association of *TIM‐1* rs9313422 G>C and rs41297579 G>A polymorphisms with the risk of CAP in children

Hardy‐Weinberg equilibrium (HWE) test showed that the genotype frequencies of *TIM‐1* rs9313422 G>C and rs41297579 G>A in the control group were in genetic equilibrium, indicating that the sample analyzed was representative of the population (Table 2). Besides, as illustrated in Table 3, rs9313422 G>C polymorphism was related to the risk of CAP in children [codominant model: CC vs GG, OR (95%CI) = 3.125 (1.500‐6.512), *P* = .002; dominant model: GC + CC vs GG, OR (95%CI) = 2.111 (1.237‐3.602), *P* = .006; recessive model: CC vs GC + GG, OR (95%CI) = 2.378 (1.214‐4.659), *P* = .010; and allele model: C vs G, OR (95%CI) = 1.938 (1.328‐2.829), *P* = .001]. In addition, the A allele of rs41297579 G>A could increase the risk of CAP in children [allele model: A vs G, OR (95%CI) = 1.727 (1.073‐2.778), *P* = .023]. According to the SHEsis analysis, the haplotype GA (rs9313422‐rs41297579) and GG can significantly reduce the risk of CAP in children [GA: OR (95%CI) = 0.380 (0.174‐0.835), *P* = .013; GG: OR (95%CI) = 0.688 (0.477‐0.993), *P* = .046]; while the haplotype CA would significantly elevate children's risk of CAP [OR (95%CI) = 4.811 (2.406‐9.622), *P* < .001] (Table 4).

**Table 2:** Hardy‐weinberg equilibrium of rs9313422 G>C and rs41297579 G>A in case group and control group

Genotype	Case group (n = 112)				Health controls (n = 120)			
	Theoretical value	Practical value	*χ* ^2^	*P*	Theoretical value	Practical value	*χ* ^2^	*P*
rs9313422 G>C								
GG	31.08	36			56.03	60		
GC	55.84	46			51.93	44		
CC	25.08	30	3.477	.062	12.03	16	2.800	.094
rs41297579 G>A								
GG	66.81	71			87.55	89		
GA	39.39	31			29.90	27		
AA	5.81	10	5.080	.024	2.55	4	1.126	.289

**Table 3:** Association of *TIM‐1* rs9313422 G>C and rs41297579 G>A polymorphisms with the risk of children CAP in children

Model	rs9313422 G>C					rs41297579 G>A				
	Case group (n = 112)	Health controls (n = 120)	χ^2^	*P*	OR (95%CI)	Case group (n = 112)	Health controls (n = 120)	χ^2^	*P*	OR (95%CI)
Codominant										
1/1	36 (32.14%)	60 (50.00%)				71 (63.69%)	89 (74.17%)			
1/2	46 (41.07%)	44 (36.67%)	3.491	.062	1.742 (0.971‐3.126)	31 (27.68%)	27 (22.50%)	1.408	.236	1.439 (0.788‐2.630)
2/2	30 (26.79%)	16 (13.33%)	9.604	.002	3.125 (1.500‐6.512)	10 (8.93%)	4 (3.33%)	3.787	.052	3.134 (0.943‐10.420)
Dominant										
1/1	36 (32.14%)	60 (50.00%)				71 (63.69%)	89 (74.17%)			
½ + 2/2	76 (67.86%)	60 (50.00%)	7.616	.006	2.111 (1.237‐3.602)	41 (36.61%)	31 (25.83%)	3.142	.076	1.658 (0.946‐2.906)
Recessive										
1/1 + ½	82 (73.21%)	104 (86.67%)				102 (91.07%)	116 (96.67%)			
2/2	30 (26.79%)	16 (13.33%)	6.595	.010	2.378 (1.214‐4.659)	10 (8.93%)	4 (3.33%)	3.198	.074	2.843 (0.865‐9.345)
Overdominant										
1/1 + 2/2	66 (58.93%)	76 (63.33%)				81 (72.32%)	93 (77.50%)			
½	46 (41.07%)	44 (36.67%)	0.473	.491	1.204 (0.709‐2.043)	31 (27.68%)	27 (22.50%)	0.829	.363	1.318 (0.726‐2.392)
Allele model										
1	118 (52.68%)	164 (68.33%)				173 (77.23%)	205 (85.42%)			
2	106 (47.32%)	76 (31.67%)	11.910	.001	1.938 (1.328‐2.829)	51 (22.77%)	35 (14.58%)	5.140	.023	1.727 (1.073‐2.778)

Abbreviations: OR, odds ratio; 95% CI, 95% confidence intervals; 1/1, Homozygous wild type; 1/2, Heterozygous; 2/2, Homozygous mutant.

**Table 4:** Haplotype association analysis to CAP children using the two SNPs (rs9313422 G>C ‐ rs41297579 G>A)

rs931342 ‐ rs41297579	Case (freq)	Control (freq)	*χ* ^2^	*P*	OR	95%CI
CA	41.89 (0.187)	10.95 (0.046)	22.949	<.001	4.811	2.406‐9.622
CG	63.11 (0.282)	65.05 (0.271)	0.066	.797	1.055	0.702‐1.585
GA	9.11 (0.041)	24.05 (0.100)	6.188	.013	0.380	0.174‐0.835
GG	109.89 (0.491)	139.95 (0.583)	3.993	.046	0.688	0.477‐0.993

Abbreviations: 95% CI, 95% confidence intervals; OR, odds ratio.

### 3.4 Association of *TIM‐1* rs9313422 G>C and rs41297579 G>A polymorphisms with the clinical characteristics of CAP in children

Stratified analysis was carried out based on the clinical characteristics of CAP in children (Table 5), and the result demonstrated that under the dominant model, both rs9313422 G>C and rs41297579 G>A polymorphisms were related to the severity of CAP in children (all *P* < .05), but they had no correlations with gender, identified pathogens and mortality of children with CAP (all *P* > .05). In addition, rs9313422 G>C had an impact on the ICU admission of patients (*P* < .05).

**Table 5:** Association of *TIM‐1* rs9313422 G>C and rs41297579 G>A polymorphisms with the clinical characteristics of CAP in children

Characteristics	rs9313422 G>C			rs41297579 G>A		
	GG (n = 36)	GC + CC (n = 76)	*P*	GG (n = 71)	GA + AA (n = 41)	*P*
Gender						
Male	22 (19.64%)	42 (37.50%)		41 (36.61%)	23 (20.54%)	
Female	14 (12.50%)	34 (30.36%)	.559	30 (26.79%)	18 (16.07%)	.865
Identified pathogens						
Bacterial	5 (4.46%)	6 (5.36%)		6 (5.36%)	5 (4.46%)	
Viral	3 (2.68%)	6 (5.36%)		7 (6.25%)	2 (1.79%)	
Bacterial and viral	2 (1.79%)	1 (0.89%)		1 (0.89%)	2 (1.79%)	
Yeast	2 (1.79%)	1 (0.89%)		3 (2.68%)	0 (0.00%)	
Unknown	25 (22.32%)	61 (54.46%)	.572	54 (48.21%)	32 (28.57%)	.394
Pneumonia severity						
Mild	20 (17.86%)	17 (15.18%)		31 (27.68%)	6 (5.36%)	
Moderate	10 (8.93%)	31 (27.68%)		23 (20.54%)	18 (16.07%)	
Severe	6 (5.36%)	28 (25.00%)	.002	17 (15.18%)	17 (15.18%)	.006
ICU admission						
No	15 (13.39%)	14 (12.50%)		22 (19.64%)	7 (6.25%)	
Yes	21 (18.75%)	62 (55.36%)	.009	49 (43.75%)	34 (30.36%)	.105
Mortality						
No	34 (30.36%)	72 (64.29%)		68 (60.71%)	38 (33.93%)	
Yes	2 (1.79%)	4 (3.57%)	.949	3 (2.68%)	3 (2.68%)	.484

### 3.5 Relationship between *TIM‐1* rs9313422 G>C and rs41297579 G>A polymorphisms and inflammatory responses of CAP in children

**Figure 3:**
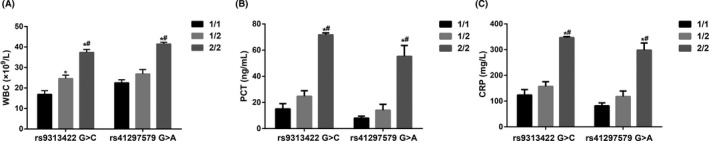
Relationship of between *TIM‐1* rs9313422 G>C and rs41297579 G>A polymorphisms with and inflammatory responses of CAP in children. Note: 1/1, Homozygous wild type; 1/2, Heterozygous; 2/2, Homozygous mutant; OR, Odds Ratio; 95% CI, 95% Confidence Intervals; ^*^, *P* < .05 compared with homozygous wild‐type carriers; ^#^, *P* < .05 compared with heterozygous genotype carriers

As presented in Figure 3, CAP patients who carried with the mutant genotypes of rs9313422 G>C (GC and CC) had remarkably higher in WBC than GG genotype carriers (all *P* < .05); meanwhile, the AA genotype carriers in rs41297579 G>A revealed a higher WBC, as compared with the GG and GC genotype carriers (all *P* < .05). However, there was no significant difference in terms of WBC between patients carrying GG genotype and those carrying GC genotypes of rs9313422 G>C (*P* > .05). Moreover, the levels of PCT and CRP were statistically higher in patients carrying the homozygous mutant of rs9313422 G>C and rs41297579 G>A than those of corresponding homozygous wild type and heterozygous carriers (all *P* < .05), but no significant difference was observed between homozygous wild‐type carriers and heterozygous genotype carriers with respect to the PCT and CRP levels (all *P* > .05).

## 4 DISCUSSION

In recent years, a large body of studies has found that the genetic polymorphisms of *TIM‐1* were correlated with the risk of a variety of diseases, including asthma,^20^ hepatitis C persistence,^21^ childhood atopic dermatitis,^22^ and systemic lupus erythematosus (SLE).^23^

In this study, we analyzed two SNPs in the human promoter region of *TIM‐1* gene by performing genotyping via PCR‐RFLP, and we found that the C allele of rs9313422 G>C and the A allele of rs41297579 G>A were significantly associated with the increased risk of CAP in children. Consistent with our finding, these two SNPs were also demonstrated to be related to the susceptibility of allergic rhinitis, as suggested by Mou et al^24^ Also, Shirzade et al and Chen et al reported that the gene polymorphisms of *TIM‐1* rs9313422 G>C may function as a predisposing factor for atopic asthma and childhood allergic asthma.^16^, ^17^ In the study by Xie et al, the rs9313422 CC genotype and rs41297579 AA genotype could greatly affect the susceptibility and prognosis of dilated cardiomyopathy,^11^ indicating that the gene mutation of rs9313422 G>C and rs41297579 G>A might be a risk factor for multiple diseases along the above evidence, as well as in children with CAP. As we know, both of the two SNPs were located in the promoter region of *TIM‐1*,^25^ and there was evidence stating that the polymorphisms within promoter regions couldnot change the coding sequence of a specific gene, but they could possibly influence the initiation and promotion of gene transcription, thereby exerting pathogenic effects.^11^ More importantly, TIM‐1 was widely accepted as an important pathogenic factor of inflammatory diseases, including pneumonia,^11^ which can be selectively expressed on the surface of activated CD4^+^ T cells, thus promoting the secretion of Th2 cytokines, stimulating the proliferation and activation of B cells, and promoting the production of antibodies (IgE and IgA).^11^, ^26^ Furthermore, we used the online software SHEsis to carry out haplotype analysis, and the result demonstrated both haplotypes GA and GG could effectively reduce the risk of CAP in children. As such, we hypothesized that the *TIM‐1* promoter haplotype together with T‐cell receptor can induce the production of anti‐inflammatory Th2 cytokines, and thus having a protective effect on CAP; while CA genotype can significantly increase the risk of childhood CAP.

As one of the most common community‐acquired infections, CAP usually means the necessity of ICU admission.^27^ Severe CAP is a major cause of patients’ mortality, and about 12%‐36% CAP patients in the ICU die within a short period despite the application of effective antibiotic therapies.^28^ To date, numerous studies have proved the association of gene polymorphisms with the severity and prognosis of CAP patients. For instance, Gallagher et al observed a significant increase in the G allele frequency of IL‐10 in CAP patients with the increase in illness severity.^29^ In the study by Yende et al, the existence of C allele at the −173 G/C position of macrophage migration inhibitory factor (*MIF*) gene was relevant to the higher 90‐d survival of CAP patients.^30^ In the current research, we found that under the dominant model, both rs9313422 G>C and rs41297579 G>A were related to the severity of childhood CAP, and rs9313422 G>C was also associated with patients’ admission rate of ICU, which highlighted the impacts of these two SNPs on the progression and prognosis of CAP in children. Besides, it was also worthy to mention that the patients who carried with the mutant genotypes of rs9313422 G>C and rs41297579 G>A showed a non‐statistically higher mortality rate than those wild‐type genotype carriers, which may be attributed to the small sample size to some extent. In addition, a wide range of studies have pointed out PCT, CRP, and WBC as three crucial inflammatory markers in CAP, since TIM‐1 was identified to have a relation with many inflammatory conditions.^31^, ^32^ Of note, gene polymorphisms may affect the expression of these markers. In the study by Li and his group, the frequency of rs2239185 TT genotype in vitamin D receptor (VDR) gene was significantly higher in CAP with WBC > 10×10^9^/L, CRP > 25 mg/L, and PCT > 2 ng/mL, indicating that it was correlated with the severity of CAP.^7^ As such, our study also determined the expressions of PCT, CRP and WBC in the two SNPs and found that carriers with mutant homozygotes of rs9313422 G>C and rs41297579 G>A showed statistically higher in WBC, PCT, and CRP than those carrying wild type and heterozygous genotypes, showing that CAP children with C allele of rs9313422 and A allele of rs41297579 may manifest more severe inflammatory responses, and consequently affecting the disease severity and prognosis of these children.

In summary, the C allele of rs9313422 G>C and A allele of rs41297579 G>A in the promoter region of *TIM‐1* could increase the risk of CAP in children, which were associated with the inflammatory responses and disease severity. Besides, the haplotypes GA (rs9313422‐rs41297579) and GG could reduce the risk of CAP in children, while CA could elevate the risk.

## AUTHOR CONTRIBUTION

Yang Liu designed the study, analyzed the data, and wrote the manuscript. Hong‐Bo Xu conducted the experiment, analyzed the data, and revised the manuscript. All of them approved the final manuscript.
